# TRX2/Rab35 Interaction Impairs Exosome Secretion by Inducing Rab35 Degradation

**DOI:** 10.3390/ijms23126557

**Published:** 2022-06-12

**Authors:** Tao Zhang, Lili Zhao, Liping Han, Yan Li, Lanlin Hu, Huani Wang, Fangdong Zou

**Affiliations:** College of Life Sciences, Sichuan University, Chengdu 610065, China; nzhangtao@163.com (T.Z.); zllblue@126.com (L.Z.); hanliping0204@163.com (L.H.); 18054713505@163.com (Y.L.); hulanlin@yahoo.com (L.H.); haohaowang7@163.com (H.W.)

**Keywords:** TRX2, Rab35, protein–protein interaction, exosome, mitochondria, migration

## Abstract

Given that exosomes mediate intercellular communication by delivering cellular components to recipient cells or tissue, they have the potential to be engineered to deliver therapeutic payloads. However, the regulatory mechanism of exosome secretion is poorly understood. In addition, mitochondrial components have been found in exosomes, suggesting communication between mitochondria and exosomes. However, the molecular mechanism of the mitochondria and vesicle interaction remains unclear. Here, we showed that mitochondrial thioredoxin 2 (TRX2) decreased exosome concentrations and inhibited HCT116 cell migration. Coimmunoprecipitation/mass spectrometry (Co-IP/MS) showed that TRX2 interacted with Rab35. TRX2 and Rab35 bound to each other at their N-terminal motifs and colocalized on mitochondria. Furthermore, TRX2 induced Rab35 degradation, resulting in impaired exosome secretion. Additionally, Rab35 mediated the suppressive effects of TRX2 on cell migration, and TRX2 suppressed cell migration through exosomes. Taken together, this study first found an interaction between TRX2 and Rab35. These results revealed a new role for TRX2 in the regulation of exosome secretion and cell migration and explained the upstream regulatory mechanism of Rab35. Furthermore, these findings also provide new molecular evidence for communication between mitochondria and vesicles.

## 1. Introduction

The development and progression of tumors are accompanied by the release of numerous exosomes [1,2], which suggests the potential function of exosomes in the tumor microenvironment. Exosomes, which are heterogeneous vesicles 30–150 nm in diameter that originate from endosomes, are derived from the intraluminal vesicles (ILVs) of multivesicular bodies (MVBs) and are secreted into the extracellular matrix (ECM) [3,4]. Exosomes contain some cytosolic components of their cells of origin, including an abundance of nucleic acids, proteins, lipids, and other analytes [5]. It has been proposed that exosomes mediate intercellular communication, particularly in the context of immune responses, cardiovascular diseases, and cancer, thus affecting normal and pathological conditions [3,6]. Exosomes influence neoplasia [7,8], tumor growth [9], metastasis [10,11], angiogenic and extracellular matrix remodeling [12], and resistance to chemotherapy [13]. Exosomes can be used not only to diagnose various pathologies, but also to deliver therapeutic drug payloads, including short interfering RNAs, antisense oligonucleotides, chemotherapeutic agents, and immune modulators, to a desired target [5,14]. Thus, it is particularly vital to understand the biogenesis and secretion mechanisms of exosomes.

Exosome biogenesis mainly depends on ESCRT (endosomal complex required for transport) proteins [3,15]. Rab proteins are essential regulators of exosome secretion [3,16]. Rab35 and its GTPase-activating proteins, TBC1D10A–C, regulate PLP-bearing exosome secretion [17]. Rab27a/b and their known effectors, SYTL4 and EXPH5, are involved in multivesicular endosome (MVE) docking at the plasma membrane to promote exosome secretion [18]. Rab11 mediates the tethering/docking of MVBs to promote homotypic fusion in a calcium-dependent manner [19]. Although several regulators are involved in exosome secretion, mitochondrial proteins have not been reported.

Oncogenic Rab35, a member of the Ras-related protein GTPase family, acts as a molecular switch by exchanging between its active GTP-bound form and inactive GDP-bound form [20,21]. Rab35 is required for multivesicular motion and docking to the plasma membrane [22,23]. It has been reported that Rab35 plays an important role in several cell activities, including exosome release [17,24], endosome membrane transport [25], cytokinesis [26], and cell migration [27]. However, the upstream regulators of Rab35 are still unclear.

As an important energy factory, mitochondria are semiautonomous in cells [28,29]. Studies have shown that there are interactions between mitochondria and other membrane-coated organelles, such as the nucleus, endoplasmic reticulum, peroxisomes, lysosomes [29], and vesicles [30,31,32,33,34]. The VDAC-GRAP75-IP3R complex provides Ca^2+^ channels between the endoplasmic reticulum (ER) and mitochondria to maintain organelle Ca^2+^ homeostasis [35]. MAPL, an outer membrane mitochondria-anchored protein ligase, promotes mitochondrial fragmentation and leads to the formation of mitochondria-derived vesicles (MDVs) [36]. MAPL-containing MDVs carry TOM20, and certain specific metabolites are targeted to peroxisomes [36]. MDVs can also transport damaged mitochondria to lysosomes for degradation by mitophagy [30,31], and mitochondrial components (e.g., mtDNA) are found in extracellular vesicle exosomes [32,33,34], suggesting communication between mitochondria and vesicles. However, the molecular mechanism of mitochondria and vesicle communication remains unclear.

The mitochondrial membrane protein TRX2 (also known as TRX2) is a member of the thioredoxin (TRX) family, which shares a similar active motif: Cys-Xxx-Xxx-Cys [37,38]. The TRX system, which consists of thioredoxin, thioredoxin reductase (TRXR), and reduced NADPH, is a key antioxidant system that regulates the redox status [39]. Members of the TRX family in humans mainly include cytosolic TRX1 and mitochondrial TRX2 [37,39]. Recently, there have been many studies on the role of the TRX system in the development and progression of cancer [40,41]. The results have shown that the thioredoxin system plays important roles in the development of various tumors and drug resistance [39,41]. Beyond that, whether thioredoxin might have new features or other target proteins needs to be explored.

This study demonstrated that the mitochondrial protein TRX2 interacted with Rab35 and induced Rab35 ubiquitination and degradation, resulting in impaired exosome secretion. These data revealed a new role for TRX2 in the regulation of exosome secretion and the upstream regulatory mechanism of Rab35. These results also provided new molecular evidence of communication between mitochondria and vesicles.

## 2. Results

### 2.1. TRX2 Plays a Role in Exosome Secretion and Cell Migration

**Figure 1:**
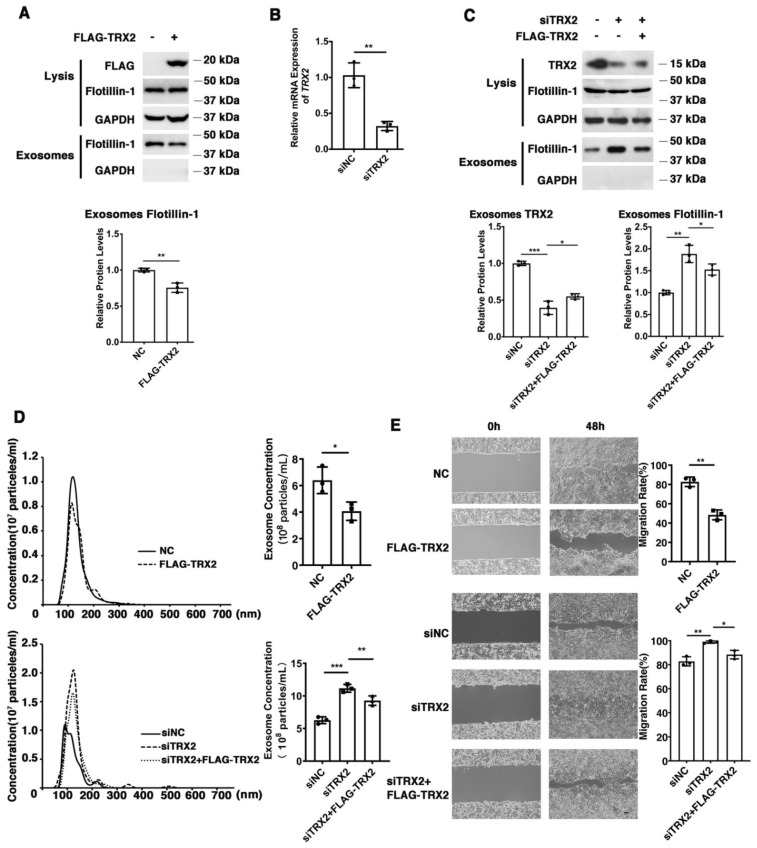
TRX2 plays a role in exosome secretion and cell migration in HCT116 cells. (**A**) HCT116 cells were transfected with pcDNA3.1+ or FLAG−TRX2. After 72 h, exosome pellets were resuspended in the corresponding volume of PBS according to cell numbers to ensure that the same volume of PBS contained exosomes from the same cell number. The lysis (20 μg) and exosomes (20 μL) proteins were detected by WB. (**B**) HCT116 cells were transfected with siNC, siTRX2−247, or siTRX2, plus low levels of siRNA−resistant FLAG−TRX2. After 48 h, the transcription level of *TRX2* was detected by real−time quantitative PCR. (**C**) HCT116 cells were transfected with siNC or siTRX2. After 72 h, exosome pellets were resuspended in the corresponding volume of PBS according to cell numbers to ensure that the same volume of PBS contained exosomes from the same cell number. The lysis (20 μg) and exosome (20 μL) proteins were detected by WB. (**D**) HCT116 cells were transfected with pcDNA3.1+, FLAG−TRX2, siNC, siTRX2, or siTRX2, and the FLAG−TRX2 EVs (extracellular vesicles) were isolated from the 10 mL cell culture supernatants of 50 million cells by serial ultracentrifugation and resuspended in 100 μL PBS. Exosome size and particle concentration were analyzed using NS300 nanoparticle characterization system. (**E**) HCT116 cells were transfected with pcDNA3.1+, FLAG−TRX2, siNC, siTRX2, or siTRX2, plus the FLAG−TRX2, and migration was measured by a scratch test. NC: pcDNA3.1+ as negative control; siNC: siRNA negative control. The scale bar = 50 μm. The experiments were performed for three biological replicates. The error bars represent means ± SD. * *p* < 0.05, ** *p* < 0.01, and *** *p* < 0.001 using the *t* test.

To explore the effect of TRX2 on colorectal cancer (CRC) cells, we overexpressed or knocked down TRX2 in HCT116 cells and evaluated the efficiency of overexpression plasmid FLAG-TRX2 and the siRNA targeting TRX2 by detecting the cell lysis (Figure 1A–C). To avoid off-target effects, we rescued the knockdown of TRX2 expression by low levels of siRNA-resistant FLAG-TRX2. The expression of cellular Flotillin-1 (an exosomal biomarker) was hardly changed, but the exosomal Flotillin-1 was decreased in TRX2-overexpressing HCT116 cells (Figure 1A). The expression of exosomal Flotillin-1 was increased when TRX2 was knocked down with siTRX2-247 and rescued by the FLAG-TRX2 (Figure 1C). As shown in Figure 1D, the exosome (Supplementary video) concentration was decreased after overexpressing TRX2 in HCT116 cells, but increased after TRX2 knockdown, which could be rescued when overexpressing TRX2. In addition, the migration rate significantly decreased after overexpressing TRX2 in HCT116 cells, whereas the migration rate significantly increased after knocking down TRX2 (Figure 1E). These results suggested that TRX2 might impair exosome secretion and suppress CRC cell migration.

### 2.2. TRX2 Interacts with Rab35, and They Colocalize on Mitochondria

**Figure 2:**
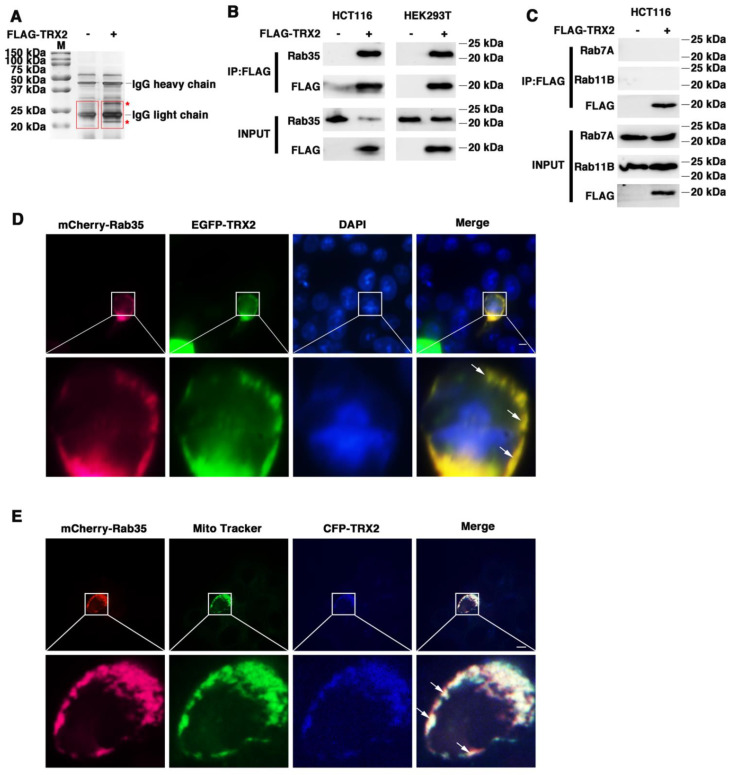
TRX2 interacted with Rab35 and they colocalized on mitochondria. (**A**) Silver staining. IP was performed with anti−FLAG magnetic beads. The * represents the specific protein bands of HCT116 cells transfected with FLAG−TRX2. The red boxes represent the gel piece excised for mass spectrometry. (**B**) HCT116 and HEK293T cells were transfected with pcDNA3.1+ or FLAG−TRX2. After 72 h, coimmunoprecipitation was performed, and Rab35 was detected. (**C**) HCT116 cells were transfected with pcDNA3.1+ or FLAG−TRX2. After 72 h, coimmunoprecipitation was performed, and Rab7A and Rab11B were detected. (**D**) HCT116 cells were transfected with EGFP−tagged TRX2 and mCherry−tagged Rab35. Rab35 (magenta), TRX2 (green), and nuclei (blue) are shown separately or merged. The scale bar = 10 μm. Magnification (lower panels) was ×5. (**E**) HCT116 cells were transfected with mCherry−tagged Rab35 or both mCherry−tagged Rab35 and CFP−tagged TRX2. After 48 h, the mitochondria were labelled with MitoTracker Green. Rab35 (magenta), mitochondria (green), and TRX2 (blue) are shown separately or merged. The experiments were performed for three biological replicates (indicated by arrows). The scale bar = 10 μm. Magnification (lower panels) was ×5.

Proteins often do not function individually, but rather as a team in a dynamic network [42]. Protein–protein interactions are central to most biological processes—from intercellular communication to programmed cell death—and therefore represent important mechanism of protein networks [43]. To further confirm the mechanism of TRX2, coimmunoprecipitation/mass spectrometry (Co-IP/MS) technology was used to identify proteins that interact with TRX2. Immunoprecipitation was conducted to pull down the fusion protein FLAG-TRX2 using anti-FLAG beads (Figure S1), followed by the silver staining of proteins. There were several protein bands specific to the FLAG-TRX2 case (shown with a red * in Figure 2A). Further mass spectrometry analysis of the specific bands (shown in the red box) revealed 32 proteins in the FLAG-TRX2 sample (Table S1). Among these 32 proteins, three Rab GTPase proteins, Rab7A, Rab11B, and Rab35, have been reported to participate in vesicle trafficking (17, 21). Therefore, we further verified the interaction between the three Rab GTPase proteins and TRX2 in HCT116 and HEK293T cells.

HCT116 cells were transfected with FLAG-TRX2, and the cell lysates were subjected to preclearance with control IgG and then incubated with anti-FLAG antibodies for IP. The IP products were then blotted with anti-Rab35, anti-Rab7A, or anti-Rab11B. As shown in Figure 2B, the immunoprecipitation of TRX2 with the anti-FLAG antibody pulled down not only TRX2, but also Rab35 in HCT116 cells and HEK293T cells. However, immunoprecipitating TRX2 did not pull down either Rab7A or Rab11B (Figure 2C). The fluorescent staining of TRX2 and Rab35 in HCT116 cells transiently transfected with EGFP-TRX2 and mCherry-Rab35 showed considerable colocalization of TRX2 (green) and Rab35 (magenta) (Figure 2D). Furthermore, the fluorescent staining of Rab35 (magenta) and mitochondria (green) indicated that Rab35 was distributed throughout the cells, but hardly located on mitochondria (Figure 2E). The fluorescent staining of TRX2, Rab35, and mitochondria in HCT116 cells transiently transfected with CFP-TRX2 and mCherry-Rab35 revealed the subcellular localization of TRX2 (blue) and Rab35 (magenta) on mitochondria (green) (Figure 2E). The IP and fluorescence results collectively confirmed that TRX2 interacted with Rab35 and that they colocalized on mitochondria.

### 2.3. TRX2 and Rab35 Bind to Each Other through Their N-Terminal Motifs

**Figure 3:**
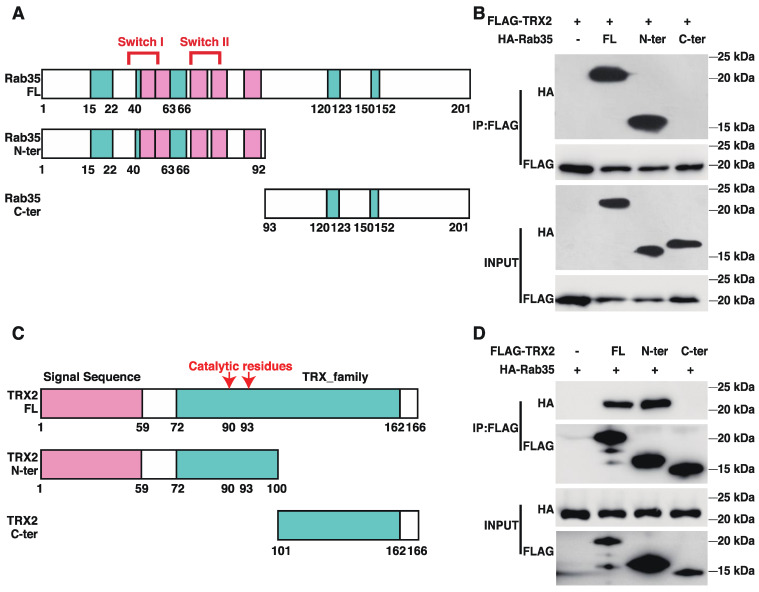
TRX2 and Rab35 bound to each other through N−terminal motifs. (**A**) Schematic diagram of Rab35. Amino acids 33 and 38–46 are switch I domain, and amino acids 66 and 68–78 are switch II domain. Green indicates the G box and pink indicates RABF, the Rab family motif. Amino acids 1–92 are the truncated N−terminal (N−ter) region of Rab35, which contains the switch domain. Amino acids 93–201 are the truncated C−terminal (C−ter) domain of Rab35. (**B**) HCT116 cells were transfected with FLAG−tagged full−length (FL) TRX2 and HA−tagged full−length Rab35, Rab35 N−ter, or Rab35 C−ter. After 72 h, coimmunoprecipitation was performed with anti−FLAG beads. The pellet was analyzed by WB. (**C**) Schematic diagram of TRX2. In the pink region, amino acids 1–59 represent the signal peptide (to mitochondria). In the green region, amino acids 72–162 represent the thioredoxin family domains. The 90th and 93rd amino acids are the catalytic active sites of TRX2. The 1st to 100th amino acids are the truncated N−terminal (N−ter) region of TRX2, which contains the signal sequence and catalytic active sites. (**D**) HCT116 cells were transfected with HA−flagged Rab35 and FLAG−tagged full−length TRX2, TRX2 N−ter, or TRX2 C−ter. After 72 h, coimmunoprecipitation was performed with anti−FLAG beads. The pellets were analyzed by WB. The experiments were performed for three biological replicates.

To identify the interactive regions of TRX2 and Rab35, we generated truncated versions of TRX2 and Rab35 (Figure 3A,C). The pulldown TRX2 from the lysates of HCT116 cells coexpressing FLAG-tagged full-length (FL) TRX2 and HA-tagged full-length Rab35, its N-terminal motif (amino acids 1–92, containing the switch I and II domains), or its C-terminal motif (amino acids 93–201) revealed an interaction between TRX2 and the N-terminus of Rab35 and the full-length Rab35 protein (Figure 3B). Meanwhile, the immunoprecipitation of full-length (FL) TRX2, the N-terminal motif (amino acids 1–100, including the signal sequence and catalytic region), and the C-terminal motif (amino acids 101–166) from the lysates of HCT116 cells coexpressing HA-tagged full length (FL) Rab35 and FLAG-tagged FL TRX2 or truncated TRX2 demonstrated the interaction between Rab35 and the N-terminus of TRX2 and the full-length TRX2 protein (Figure 3D). These data indicated that TRX2 and Rab35 interacted with each other at the N-terminus of each protein.

### 2.4. TRX2 Induces Rab35 Ubiquitination and Degradation

**Figure 4:**
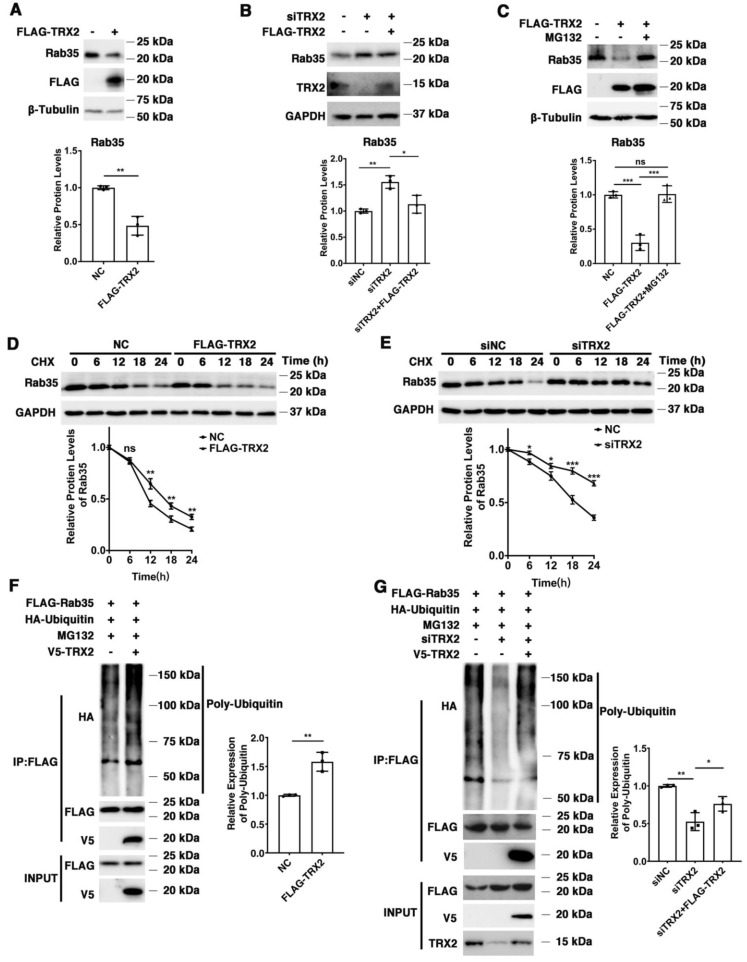
TRX2 induced Rab35 ubiquitination and degradation. (**A**) HCT116 cells were transfected with pcDNA3.1+ or FLAG−TRX2. Rab35 was detected by WB. (**B**) HCT116 cells were transfected with siNC, siTRX2, or siTRX2, plus low levels of FLAG−TRX2. Rab35 was detected by WB. (**C**) HCT116 cells were transfected with pcDNA3.1+ or FLAG−TRX2 and treated with or without MG132. Rab35 was detected by WB. (**D**) HCT116 cells were transfected with pcDNA3.1+ or FLAG−TRX2− and treated with cycloheximide (CHX) for the indicated times. Rab35 was detected by WB. (**E**) HCT116 cells were transfected with siNC or siTRX2 and treated with CHX for the indicated times. Rab35 was detected by WB. (**F**) HCT116 cells were transfected with V5−TRX2, FLAG−Rab35, or HA−tagged ubiquitin. After 48 h, the cells were treated with MG132. The ubiquitination of Rab35 was detected by IP. (**G**) HCT116 cells were transfected with FLAG−Rab35, HA−tagged ubiquitin, siTRX2, or siTRX2, plus low levels of V5−FLAG. After 48 h, the cells were treated with MG132. The ubiquitination of Rab35 was detected by IP. The experiments were performed for three biological replicates. The error bars represent means ± SD. * *p* < 0.05, ** *p* < 0.01, and *** *p* < 0.001 using the *t* test or one−way ANOVA with Šídák’s multiple comparisons test.

We found that exogenous TRX2 significantly reduced the expression of endogenous Rab35 in HCT116 cells (Figure 4A), while interference with TRX2 by siTRX2-247 upregulated Rab35 in HCT116 cells (Figure 4B). It has been reported that the E3 ubiquitination ligase mediates the ubiquitination and degradation of Rab27A [44], and TRX1 induces ASK1 degradation through ubiquitination [45]. Hence, we hypothesized that TRX2 might induce the ubiquitination and degradation of Rab35. HCT116 cells were transfected with FLAG-TRX2 and treated with or without proteasome inhibitor MG132. As shown in Figure 4C, Rab35 was significantly decreased after HCT116 cells were transfected with FLAG-TRX2, but rescued when transfected cells were treated with MG132, which suggests that TRX2 induces Rab35 degradation through the ubiquitin proteasome system. To further explore the degradation mechanism of Rab35, HCT116 cells transfected with FLAG-TRX2 or siTRX2-247 were treated with the protein synthesis inhibitor cycloheximide (CHX). Western blot analysis showed that Rab35 was obviously degraded when treated with CHX for 18 h, and this degradation was enhanced by the overexpression of TRX2 in HCT116 cells (Figure 4D). Similarly, when TRX2 was knocked down by siTRX2-247 in HCT116 cells, its degradation was inhibited (Figure 4E). Furthermore, IP experiments showed that the levels of ubiquitinated Rab35 were increased in HCT116 cells that were cotransfected with V5-TRX2, FLAG-Rab35, and HA tagged ubiquitin (HA-UB) and treated with MG132 (Figure 4F). Similarly, knocking down TRX2 by siTRX2-247 dramatically decreased the levels of ubiquitinated Rab35 (Figure 4G). Taken together, these results suggested that Rab35 was degraded mainly through the ubiquitin–proteasome pathway regulated by TRX2.

### 2.5. TRX2 Impairs Exosome Secretion Mediated by Rab35

**Figure 5:**
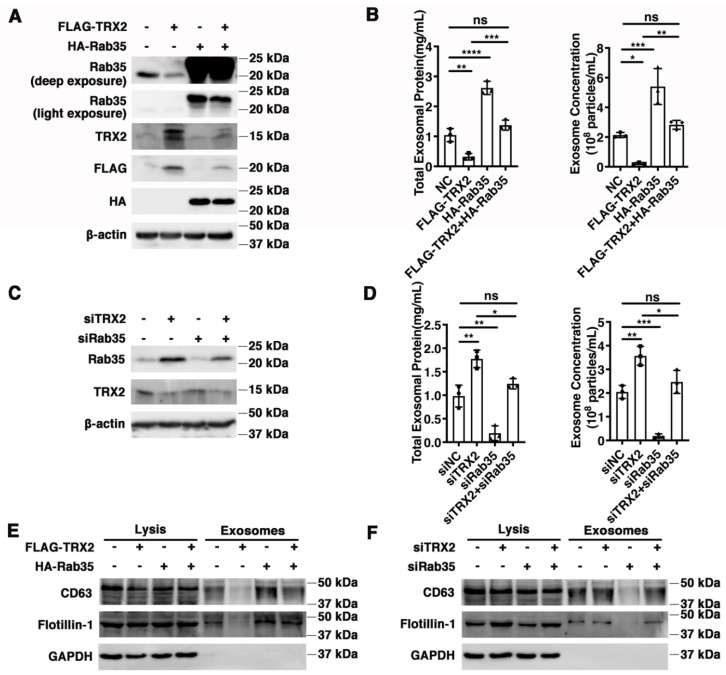
TRX2 impaired exosome secretion through Rab35 degradation. (**A**) HCT116 cells were transfected with pcDNA3.1+, FLAG−TRX2, or HA−Rab35. The indicated proteins were detected by WB. (**B**) HCT116 cells were transfected with pcDNA3.1+, FLAG−TRX2, or HA−Rab35. The EVs were isolated from the 10 mL cell culture supernatants of 25 million cells by serial ultracentrifugation and resuspended in 100 μL PBS. The total exosomal protein and exosome concentrations were measured. (**C**) HCT116 cells were transfected with siNC, siTRX2, or siRab35. The indicated proteins were detected by WB. (**D**) HCT116 cells were transfected with siNC, siTRX2, or siRab35. The EVs were isolated from the 10 mL cell culture supernatants of 25million cells by serial ultracentrifugation and resuspended in 100 μL PBS. The total exosomal protein and exosome concentrations were measured. (**E**) HCT116 cells were transfected with pcDNA3.1+, FLAG−TRX2, or HA−Rab35, and the exosomes were purified. The biomarkers of the exosomes were detected by WB in whole cell lysates (20 μg) and exosomes (20 μL). (**F**) HCT116 cells were transfected with siNC, siTRX2, or siRab35, and the exosomes were purified. The biomarkers of the exosomes were detected by WB in whole cell lysates (20 μg) and exosomes (20 μL). The experiments were performed for three biological replicates. The error bars represent means ± SD. ns, no significant difference; * *p* < 0.05, ** *p* < 0.01, *** *p* < 0.001, and **** *p* < 0.0001 using one−way ANOVA with Šídák’s multiple comparisons test.

Rab35 has been reported to regulate exosome transportation and secretion [17,24], and TRX2 was previously proven to induce Rab35 ubiquitination and degradation. Therefore, we hypothesized that exosome secretion mediated by Rab35 might be regulated by TRX2. HCT116 cells were transfected with FLAG-TRX2, HA-Rab35, or both (Figure 5A), and the exosomes were collected, purified, and analyzed. As shown in Figure 5B, the total exosomal protein and exosome concentrations were decreased in TRX2-overexpressing HCT116 cells but restored in HCT116 cells cotransfected with FLAG-TRX2 and HA-Rab35. In addition, the exosomal biomarkers Flotillin-1 and CD63 were not significantly different in cell lysis but were downregulated in the exosomes of TRX2-overexpressing HCT116 cells and recovered by Rab35 (Figure 5E). On the other hand, when TRX2 was knocked down by siTRX2-247 (Figure 5C), the total exosomal protein, exosome concentrations, and exosomal Flotillin-1 and CD63 (not a significant difference in cell lysis) were significantly upregulated (Figure 5D,F). The interference of Rab35 with siRab35 blocked the increase in exosome secretion induced by siTRX2-247 (Figure 5D,F). These results revealed that Rab35-mediated exosome secretion was inhibited by TRX2.

### 2.6. Rab35 Mediates the Suppressive Effects of TRX2 on Cell Migration

**Figure 6:**
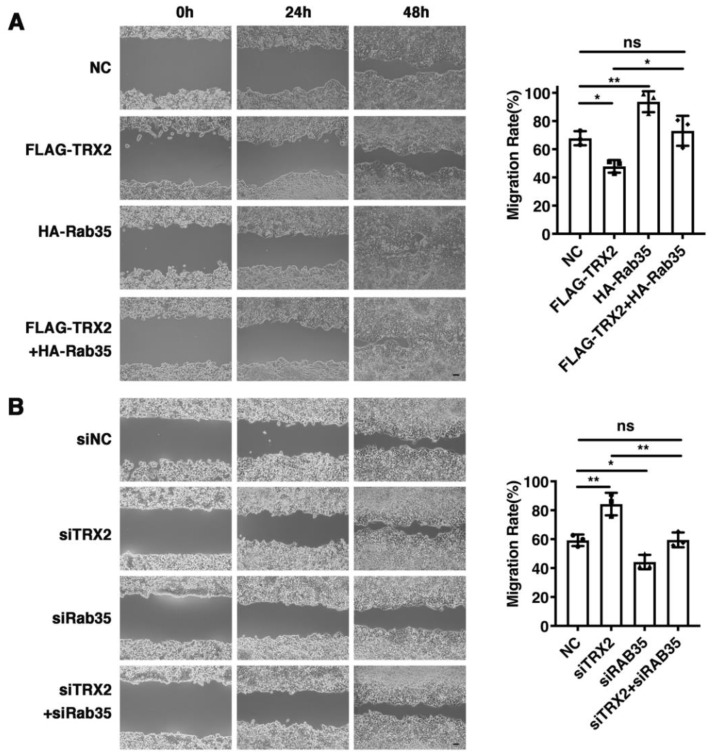
Rab35 mediated the TRX2-induced suppression of cell migration. (**A**) HCT116 cells were transfected with pcDNA3.1+, FLAG-TRX2, or HA-Rab35. Cell migration was measured by scratch tests. (**B**) HCT116 cells were transfected with siNC, siTRX2, or siRab35. Cell migration was measured by scratch tests. The experiments were performed for three biological replicates. The error bars represent means ± SD. ns, no significant difference; * *p* < 0.05 and ** *p* < 0.01 using one-way ANOVA with Šídák’s multiple comparisons test.

To explore whether TRX2 plays a role in cell migration, wound healing assays were performed. We found that the restoration of Rab35 recovered the decreased migration rate caused by TRX2 (Figure 6A). In contrast, the migration ability enhanced by siTRX2-247 was attenuated by siRab35 in HCT116 cells (Figure 6B). Collectively, these data suggested that TRX2 could hinder cell migration by degrading Rab35.

### 2.7. TRX2 Suppresses Cell Migration through Exosomes

**Figure 7:**
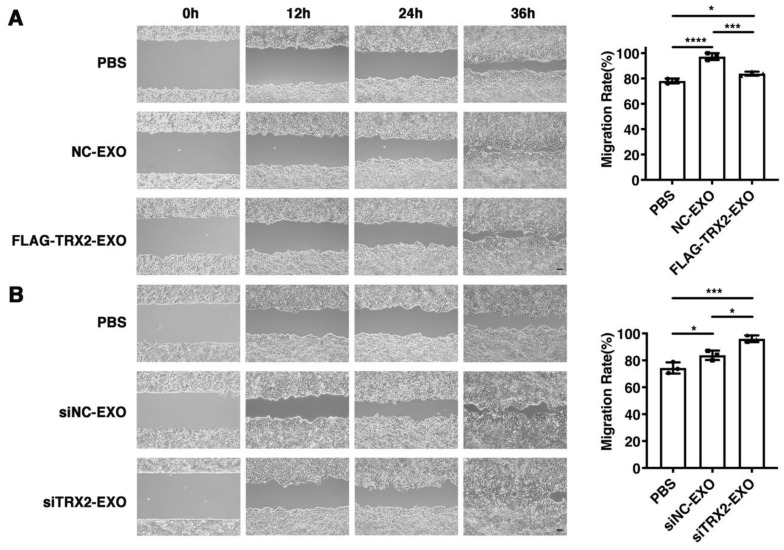
Effect of exosomes on HCT116 cell migration. (**A**) HCT116 cells were transfected with pcDNA3.1+ or FLAG-TRX2, and exosomes were purified. Then, 2 × 10^5^ particles of exosomes were added to HCT116 cells cultured in exosome-free media. Cell migration was measured by scratch tests. (**B**) HCT116 cells were transfected with siNC or siTRX2, and exosomes were purified. Then, 2 × 10^5^ particles of exosomes were added to HCT116 cells cultured in exosome-free media. Cell migration was measured by scratch tests. The experiments were performed for three biological replicates. The scale bar = 50 μm. The error bars represent means ± SD. EXO, exosome; * *p* < 0.05, *** *p* < 0.001, and **** *p* < 0.0001 using one-way ANOVA with Šídák’s multiple comparisons test.

To investigate whether TRX2 suppressed cell migration through its effect on exosome secretion, exosomes secreted from HCT116 cells transfected with the empty vector, FLAG-TRX2, siNC, or siTRX2 were purified and added to HCT116 cells cultured in exosome-free media. The wound healing assay results showed that all exosomes promoted HCT116 cell migration. However, the migration rate of HCT116 cells supplemented with exosomes from the TRX2-overexpressing cells was less than that of the HCT116 cells supplemented with exosomes from the cells transfected with the empty vector (Figure 7A). Moreover, the exosomes from TRX2-knockdown cells increased the migration rate of HCT116 cells to a greater extent than the exosomes from control cells (Figure 7B). These results suggested that TRX2 could suppress cell migration by regulating exosome secretion.

## 3. Discussion

The development and progression of tumors are regulated by both cancer cells themselves and the tumor microenvironment [46]. The tumor microenvironment, with complex matrix components, plays a vital role in tumor growth, metastasis, and occurrence [47]. The function of exosomes is to transport “cargo” and mediate extracellular communication, which depends on the precise regulation of secretion. Exosomes affect cell migration, invasion, and metastasis [1,2,47]. The endogenous properties of these vesicles allow them to survive in the extracellular space, bypass biological barriers, and deliver their biologically active molecular cargo to recipient cells. Thus, exosomes have promising therapeutic potential for the delivery of therapeutic drug payloads. It is important to understand the regulatory mechanism of exosome secretion and improve exosome yield. Although several key regulators have been reported to participate in exosome biogenesis and secretion [3], the precise regulatory mechanism is poorly understood. Rab35 plays a crucial role in exosome secretion [3,17]. The inhibition of Rab35 function leads to the intracellular accumulation of endosomal vesicles and impairs exosome secretion [17]. In our study, TRX2 impaired exosome secretion and reduced the exosome concentration by inducing Rab35 degradation, contributing to knowledge on the regulatory mechanism of exosome secretion. Therefore, TRX2 interference may be favorable to promote exosome secretion and increase exosome production. Moreover, TRX2 suppressed cell migration through Rab35 and exosomes in HCT116 cells.

Mitochondria have been recently reported to communicate with other membrane-bound organelles, such as the nucleus and lysosome [29]. Although several mitochondrial components (e.g., mtDNA) have been found in exosomes [32,33,34], communication between mitochondria and exosomes is limited, and the molecular mechanism is still unclear. Early endosomes accumulate ILVs in their lumen and mature into late endosomes, also called multivesicular endosomes or MVBs [3]. On the one hand, MVBs fuse with lysosomes for degradation. On the other hand, MVBs transport ILVs and fuse with the plasma membrane (PM) to release ILVs as exosomes [3,4,16]. In this study, we found an interaction between the mitochondrial membrane protein TRX2 and Rab35 and their roles in exosome secretion and cell migration. In addition, TRX2 and Rab35 colocalize on the mitochondrial membrane. Given that TRX2 is mainly localized on the mitochondrial membrane while Rab35 is localized at the cytosolic face of endosome-MVBs and the PM [16,23], the interaction between TRX2 and Rab35 suggests an interaction between mitochondria and endosomes. However, whether mitochondria interact with early endosomes or late endosomes (so-called MVBs) needs further proof. Additionally, TRX2 impaired exosome secretion and cell migration by degrading Rab35, which suggests a link between mitochondria and exosomes. These results provide new molecular evidence of mitochondria and vesicle communication.

TRX1 has been reported to regulate the elimination of reactive oxygen species (ROS), which promotes the development of cancer [48]. Moreover, TRX1 inhibits ASK1-mediated apoptosis by inducing ASK1 ubiquitination and degradation [45]. In addition, the subcellular localizations of TRX1 and TRX2 in cells are different. TRX1 is mainly localized in the cytoplasm, while TRX2 is mainly localized on the mitochondrial membrane. However, little is known about the function of TRX2. TRX2 is a small mitochondrial redox protein that plays a crucial role in regulating mitochondrial ROS homeostasis and early-onset neurodegeneration [49]. Redox regulation is based on the dithiol–disulfide exchange between TRX2 and its target proteins [41]. Several studies have reported the function of TRX2 in neurodevelopment, neuronal maintenance [49], angiogenesis [50], and apoptosis [51]. However, the TRX2-mediated denitrosylation of caspase-3 may promote the full activation of caspase-3 and thereby facilitate apoptosis [52,53]. Beyond that, whether TRX2 might have new functions or other target proteins needs to be explored. This study revealed that TRX2 suppressed the migration of HCT116 cells by inducing Rab35 ubiquitination and degradation and decreasing exosome concentration, which revealed a new role for TRX2 in exosome secretion. TRX1 has been reported to interact with ASK1 through its single cysteine residue (C32 or C35) to promote ASK1 ubiquitination and degradation [45], and the N-terminal motif of TRX2 also contains catalytic cysteine residues (C90, C93). We reported that TRX2 could induce Rab35 degradation might through the ubiquitination proteasomal pathway. However, further investigation is needed to identify the E3 ligase and its ubiquitin binding sites on the Rab35 protein, which will be beneficial for better understanding the posttranslational regulatory mechanisms of Rab35.

Coimmunoprecipitation/mass spectrometry (Co-IP/MS) has been widely utilized to determine protein–protein interactions. We employed Co-IP/MS to determine the proteins that interact with TRX2. We found 32 proteins that might interact with TRX2. Part of the proteins were detected probably due to nonspecific binding with magnetic beads. It would be better to purify the case with another tag antibody (e.g., Strep or GST). Further molecular experiments confirmed the interaction between TRX2 and Rab35. Co-IP showed that TXN2 interacted with Rab35 but not Rab7A or Rab11B. Different Rab GTPases are involved in different endosomal trafficking highways [54]. TXN2 might play a specific role in the exocytic trafficking highway (Rab35), but not the endomembrane commuting routes or endocytic trafficking highways (Rab7A, Rab11B). The conversion between the GDP- and GTP-bound forms of Rab GTPases involves major conformational changes in two variable regions, termed switch I and switch II. Active GTP-bound Rab mediates budding, uncoating, motility, tethering, and fusion [21]. Rab35’s functions in regulating exosome release [17,24], endosome membrane transport [25], cytokinesis [26], and cell migration [27] have been well documented, but the upstream regulatory mechanism is unclear. It has been reported that Rab35 interacts with other proteins relying on its conversion from GDP-/GTP-bound forms [20,27]. More evidence is needed to determine if TRX2 is an effector of Rab35 (i.e., whether it specifically interacts with GTP’s bound form). In the present study, we found that TRX2 and Rab35 bind to each other at the N-terminus of each protein and the N-terminal motifs of Rab35 contain the switch I and II regions, and so the interaction between TRX2 and Rab35 may also depend on GDP/GTP exchange activity. TRX1, a homolog of TRX2, has been reported to interact with ASK1 through its single cysteine residue (C32 or C35) to promote ASK1 ubiquitination and degradation. In addition, the N-terminal motif of TRX2 contains a locating signal peptide (aa 1–59) [55]. A study found that Rab35 could be recruited to mitochondria and induce mitophagy [56], indicating a link among mitochondria, Rab35, and lysosomes. In this study, we showed that TRX2 recruited Rab35 to mitochondria for ubiquitination and degradation. Accordingly, the interaction between TRX2 and Rab35 may rely on the mitochondrial targeting signal of TRX2 and catalytic cysteine residues (C90, C93).

Colorectal cancer (CRC) is one of the most common cancers in the world and is accompanied by high morbidity and mortality [57]. At the early stage, CRC can be curative, while palliative treatments are available in the advanced stages of CRC, especially when metastases are present [58]. The CRC metastasis rate has increased steadily to 20~25%, and most of these cases appear in the liver [59]. Although the 5 year overall survival rate reaches 47–60% after hepatectomy for colorectal metastases, recurrence occurs in 40~75% of patients after liver resection, and the 5 year survival rate is less than 10% with palliative chemotherapy [60]. The optimal therapeutic approach for CRC with synchronous liver metastases has not yet been clarified [60]. Accordingly, the prevention of CRC migration and metastasis would be a treatment strategy. Mutant p53-expressing tumor cells produce exosomes to gain invasive/migratory faction by controlling the production of sialomucin and podocalyxin and the activity of Rab35 GTPase [61]. This study indicates that TRX2 suppresses HCT116 colorectal cancer cell migration in a Rab35-dependent manner and that exosome-mediated TRX2 inhibition inhibits cell migration, which provides new ideas for the clinical treatment of CRC.

## 4. Materials and Methods

### 4.1. Cells, Antibodies, Primers and Reagents

The colorectal cancer cell lines HCT116 and HEK 293T cells were purchased from the Cell Bank of the Representative Culture Preservation Committee of the Chinese Academy of Sciences. Plasmids pcDNA3.1+ and pcDNA3.1/V5-HisC were purchased from Addgene. The rabbit polyclonal anti-TRX2 (13089-1-AP), rabbit polyclonal anti-Rab35 (11329-2-AP), rabbit anti-HA (51064-2-AP), rabbit anti-FLAG (20543-1-AP), rabbit anti-Flotillin-1 (15571-1-AP), and mouse anti-β-actin (60008-1-Ig) antibodies were purchased from Proteintech Group Inc. Rabbit anti-β-tubulin (2146) and rabbit anti-GAPDH (ab8245) were obtained from Cell Signaling Technology and Abcam, respectively. Anti-FLAG magnetic beads (covalently coupled with a high-quality recombinant mouse monoclonal antibody, B26101) and MG132 (S2619) were purchased from Selleck. PCR primers were synthesized by Tsingke Biotechnology Co., Ltd. (Chengdu, China), and the sequences are shown in Table S1. SiRNAs were synthesized by GenePharma (Shanghai, China), and the sequences were as follows: siTRX2-247 5′- UCU CCU UGA CAA CCU UUA ATT-3′, siRab35 5′-GCU CAC GAA GAA CAG UAA A-3′, and siNC (negative control) 5′- UUC UCC GAA CGU GUC ACG UTT-3′. The PrimeScript RT Reagent Kit with gDNA Eraser was purchased from TaKaRa (Tokyo, Japan). The real-time quantitative PCR mix was obtained from Vazyme Biotech Co., Ltd. (Nanjing, China).

### 4.2. Cell Culture and Transfection

HCT116 cells were cultured in high-glucose DMEM (HyClone, Logan, UT, USA) supplemented with 10% FBS and 1% penicillin/streptomycin at 37 °C and 5% CO_2_. Cells were transfected with plasmids and siRNAs via Lipofectamine 2000 (Thermo Fisher, Waltham, MA, USA), following the manufacturer’s protocol. After 48 h of incubation, transfection efficiency was assessed by RT-qPCR and Western blot.

### 4.3. Plasmid Construction

To tag TRX2 and Rab35, 3× FLAG or HA tags were added to the vector pcDNA3.1+. Full-length TRX2 and Rab35 were amplified from cDNA and inserted into the vectors pcDNA3.1+/3× FLAG and pcDNA3.1+/HA, respectively (named FLAG-TRX2 and HA-Rab35). The truncated sequences TRX2-1-100 aa, TRX2-101-166 aa, Rab35-1-192 aa, and Rab35-93-201 aa were amplified from FLAG-TRX2 and HA-Rab35 and inserted into the vectors pcDNA3.1+/3× FLAG and pcDNA3.1+/HA, respectively. The empty vector pcDNA3.1+ was used as the negative control (NC). To fluorescently label TRX2 and Rab35, TRX2 and Rab35 were inserted into the vectors pEGFP-N1 (green), pCFP-N1 (blue), and pmCherry-N1 (magenta). The constructed vectors were transfected into HCT116 cells.

### 4.4. Coimmunoprecipitation/Mass Spectrometry

The cells were lysed with nondenaturing RIPA buffer, followed by ultrasonication. Then, the supernatant was extracted by centrifugation at 12,000 rpm for 10 min. A portion of the supernatant was mixed with sample buffer containing β-mercaptoethanol as INPUT, and the remainder was incubated with anti-FLAG magnetic beads overnight at 4 °C. The magnetic beads were washed with PBST (PBS containing 0.5% Tween-20), and the proteins were mixed with sample buffer containing β-mercaptoethanol from the magnetic beads. The proteins were analyzed by silver staining and Western blotting. The desired gel bands were excised, and the proteins were analyzed by mass spectrometry.

The reductive alkylation of the protein was as follows: the protein was reduced by adding a final concentration of 10 mM dithiothreitol (DTT), followed by the addition of a final concentration of 55 mM ammonium iodoacetate (IAM), and, finally, 1 μg of trypsin enzyme was added for enzymatic hydrolysis overnight (8~16 h). The peptides produced by the enzymatic hydrolysis were desalted with a C18 column, drained, and dissolved in 15 μL loading buffer (0.1% formic acid and 3% acetonitrile). The peptides were analyzed on an LC-MS/MS instrument (ekspertTM NanoLC; AB Sciex TripleTOF 5600-plus) and the results were evaluated. After the LC-MS/MS was disembarked, the original disembarkation data were directly submitted to the Proteinpilot software connected to the AB SCIEX Triple TOF™ 5600 plus mass spectrometer for database retrieval. With a confidence interval of ≥95%, unique peptides of ≥1 were set and common contaminating proteins were filtered out.

### 4.5. Western Blot (WB)

The cells or exosomes were collected and lysed with RIPA buffer containing the protease inhibitor cocktail on ice. The proteins were separated by 12% SDS-PAGE and transferred onto PVDF membranes (Millipore, billerica, MA, USA). The membranes were blocked with 5% milk in TBST for 1 h at room temperature and then incubated with primary antibodies overnight at 4 °C, followed by horseradish peroxidase-conjugated secondary antibodies (1:2000). The protein bands were visualized with ECL reagent on a ChemiDoc Touch Imaging System (Bio-Rad, Hercules, CA, USA).

### 4.6. RNA Isolation and Gene Expression Analysis

The expression of specific genes was analyzed using real-time quantitative PCR. In brief, RNA was extracted from cells using an RNAiso Plus (9109, TaKaRa, Tokyo, Japan) and reverse transcribed using the PrimeScript™ RT Reagent Kit (RR047A, TaKaRa, Japan). RT–qPCR was performed on a Real Time PCR System (Bio-Rad, USA). The primer sequences for TRX2 were as follows: 5′-AGCCCGGACAATATACACCA-3′ (forward) and 5′-AATATCCACCTTG GCCATCA-3′ (reverse). The relative expression was normalized to the GAPDH level and calculated by the 2^−ΔΔCT^ method.

### 4.7. Fluorescence Colocalization

To ensure the colocalization of TRX2 and Rab35, HCT116 cells were transfected with EGFP-labelled TRX2 and mCherry-labelled Rab35. After 48 h, the nuclei were labelled with DAPI. Fluorescent images were obtained under a confocal microscope (Olympus, Tokyo, Japan). To explore their subcellular localization, HCT116 cells were transfected with CFP-labelled TRX2 and mCherry-labelled Rab35. After 48 h, the mitochondria were labelled with MitoTracker Green (C1048, Beyotime, Shanghai, China). The fluorescent images were obtained under a confocal microscope (Zeiss, Oberkoche, Germany).

### 4.8. Exosome Isolation, Characterization, and Analysis

The exosomes were purified by sequential centrifugation as previously described [44,62,63]. In brief, cells were cultured in media supplemented with 10% exosome-free FBS. After 72 h, cells were digested and counted. The supernatants were centrifuged at 500× *g* at 4 °C for 10 min to remove living cells. To remove dead cells and large cell debris, the supernatants were then centrifuged at 2000× *g* at 4 °C for 10 min. To remove large vesicles, the supernatants were centrifuged at 10,000× *g* at 4 °C for 30 min. Finally, the exosomes were harvested by centrifugation at 100,000× *g* at 4 °C for 70 min (Beckman 32 Ti rotor, Germany). The exosomes were washed with PBS (Thermo, USA) and pelleted again through ultracentrifugation at 100,000× *g* at 4 °C for 70 min. The exosome pellets were resuspended in the corresponding volume of PBS according to cell numbers to ensure that the same volume of PBS contained exosomes from the same cell number. Exosome size and particle concentration were analyzed using an NS300 nanoparticle characterization system (NanoSight, Malvern Instruments, Malvern, UK) equipped with a red laser (580 nm). The exosomes were lysed with denaturing RIPA buffer (plus the cocktail), and their markers were analyzed by WB. The exosomes were stored short-term at 4 °C and long-term at −80 °C.

### 4.9. Wound Healing Assay

A total of 50,000 cells were plated into inserts (8 mm pore size, Corning, NY, USA) in 24-well transwell plates and incubated at 37 °C for 24 h. The inserts were removed, and pictures were taken every 12 h. Three random fields were observed per well at 10× magnification, and the average migration distance per field was calculated.

### 4.10. Statistical Analysis

All statistical tests were performed with GraphPad Prism 9 software (Version 9.0.0, La Jolla, CA, USA). The intensity of each protein was quantified using ImageJ software. Biological triplicates of each experiment were performed. Comparisons between two groups were performed with the *t* test. For more than two groups, a one-way ANOVA was performed with Šídák’s multiple comparisons test. *, *p* < 0.05; **, *p* < 0.01; ***, *p* < 0.001; ****, *p* < 0.0001, and *p* > 0.05 was ns (not significant). A *p* value < 0.05 was considered statistically significant.

## 5. Conclusions

**Figure 8:**
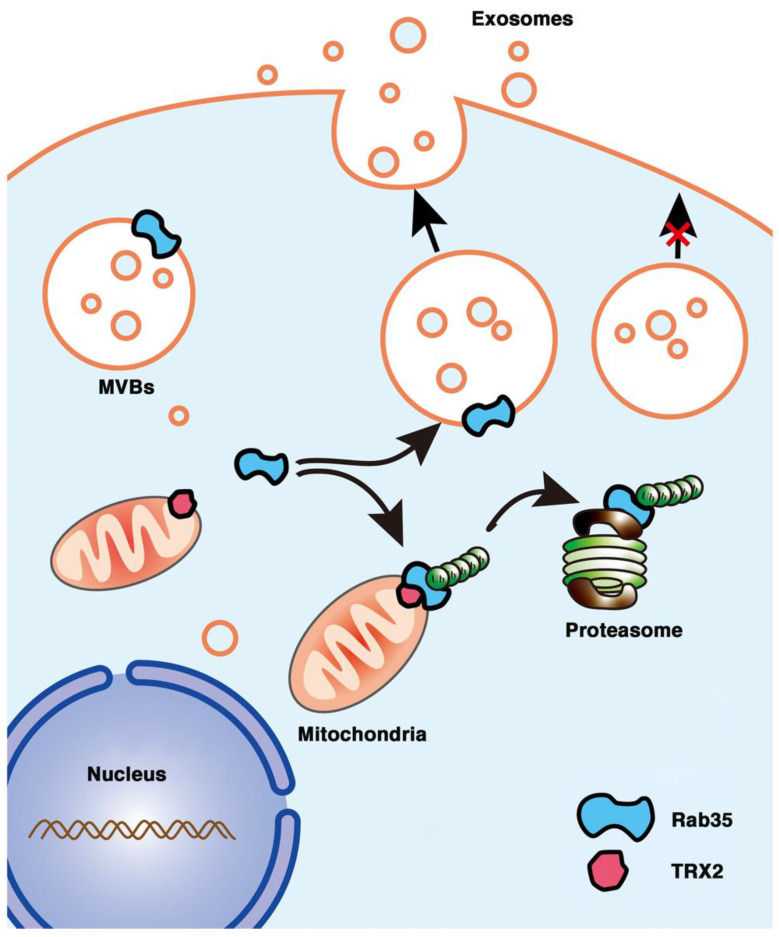
Schematic representation of the role of the TRX2/Rab35 interaction in the regulation of exosome secretion. Rab35 (at the cytosolic face of endosome-MVBs) mediates MVB docking to the cell membrane to promote exosome secretion. Mitochondrial TRX2 (on the mitochondrial membrane) interacts with Rab35 and induces Rab35 ubiquitination and degradation, resulting in impaired exosome secretion.

In conclusion, we first found that the mitochondrial protein TRX2 interacted with Rab35 at the N-terminus of each protein and recruited Rab35 to mitochondria. TRX2 induced the ubiquitination and degradation of Rab35, resulting in impaired exosome secretion and cell migration (Figure 8). The present findings indicate a new function of TRX2 in regulating exosome secretion and provide new molecular evidence for mitochondria and vesicles interaction.

## Data Availability

Not applicable.

## Supplementary Materials

The following supporting information can be downloaded at: https://www.mdpi.com/article/10.3390/ijms23126557/s1.
