# Supplementary figures and images for: TRX2/Rab35 Interaction Impairs Exosome Secretion by Inducing Rab35 Degradation
Source: Int J Mol Sci. 2022 Jun 12;23(12):6557. doi: 10.3390/ijms23126557 (PMC9224307; doi:10.3390/ijms23126557)

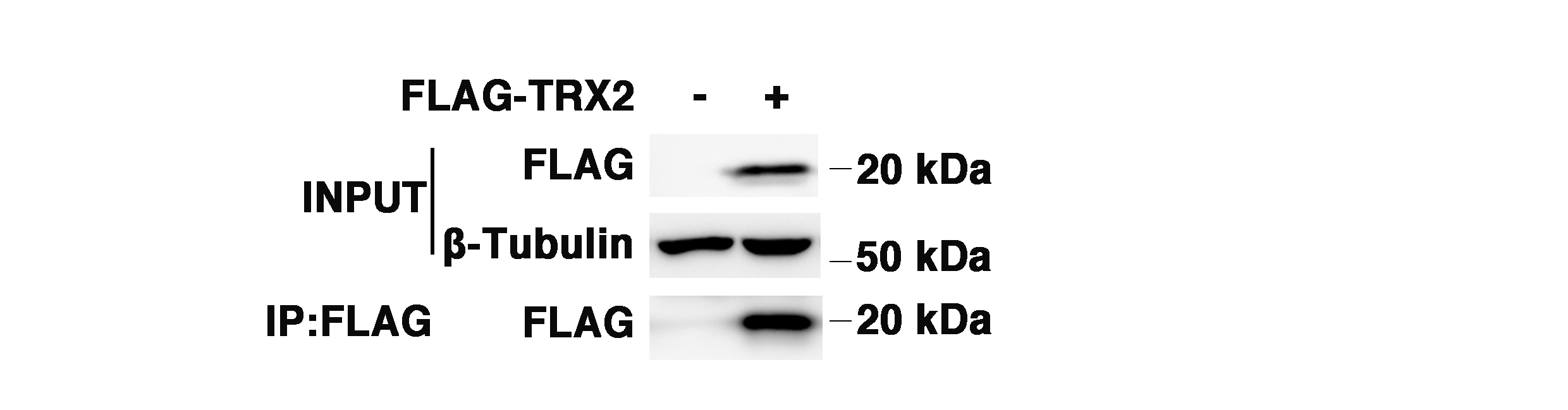

Supplement: Supplementary file 1 [file ijms-23-06557-s001.zip › S1 Detection of immunoprecipitation.tif]
